# The role of extended-spectrum cephalosporin-resistance in recurrent community-onset Enterobacteriaceae urinary tract infections: a retrospective cohort study

**DOI:** 10.1186/s12879-019-3804-y

**Published:** 2019-02-14

**Authors:** Judith A. Anesi, Ebbing Lautenbach, Irving Nachamkin, Charles Garrigan, Warren B. Bilker, Jacqueline Omorogbe, Lois Dankwa, Mary Wheeler, Pam Tolomeo, Jennifer H. Han, for the CDC Prevention Epicenters Program

**Affiliations:** 1 Division of Infectious Diseases, Department of Medicine; Center for Clinical Epidemiology and Biostatistics, Perelman School of Medicine, University of Pennsylvania, 719 Blockley Hall, 423 Guardian Drive, Philadelphia, PA 19104 USA; 2 Division of Infectious Diseases, Department of Medicine; Center for Clinical Epidemiology and Biostatistics; Department of Biostatistics, Epidemiology and Informatics, Perelman School of Medicine, University of Pennsylvania, Philadelphia, PA USA; 3 Department of Pathology and Laboratory Medicine, Perelman School of Medicine, University of Pennsylvania, Philadelphia, PA USA; 4 Center for Clinical Epidemiology and Biostatistics; Department of Biostatistics, Epidemiology and Informatics, Perelman School of Medicine, University of Pennsylvania, Philadelphia, PA USA; 5 Center for Clinical Epidemiology and Biostatistics, Perelman School of Medicine, University of Pennsylvania, Philadelphia, PA USA

**Keywords:** Community-onset, Urinary tract infection, Enterobacteriaceae, Extended-spectrum beta-lactamase (ESBL), Extended-spectrum cephalosporin-resistant

## Abstract

**Background:**

Bacterial resistance to first line antibiotics used to treat community-onset urinary tract infections (UTIs) continues to emerge. We sought to determine the association between extended-spectrum cephalosporin resistance (ESC-R) and recurrence among Enterobacteriaceae (EB) UTIs.

**Methods:**

A retrospective cohort study was performed**.** All patients presenting to the Emergency Departments (EDs) or outpatient practices in a large health system with EB UTIs between 2010 and 2013 were included. Exposed patients had ESC-R EB UTIs. Unexposed patients had ESC-susceptible EB UTIs and were matched to exposed patients 1:1 on study year. Multivariable Cox proportional hazards regression analyses were performed to evaluate the association between ESC-R EB UTI and time to recurrent UTI within 12 months.

**Results:**

A total of 302 patients with an index community-onset EB UTI were included, with 151 exposed and 151 unexposed. Overall, 163 (54%) patients experienced a recurrent UTI with a median time to recurrence of 69 days (interquartile range 25–183). On multivariable analyses, ESC-resistance was associated with an increased hazard of recurrent UTI (hazard ratio [HR] 1.39, 95% confidence interval [CI] 1.01–1.91, *P* = 0.04). Other variables that were independently associated with recurrence included a history of UTI prior to the index UTI and presence of a urinary catheter at the time of the index UTI. Secondarily, we found that when the treatment for the index UTI was adjusted for, there was no longer a significant association between ESC-R status and time to recurrent UTI (aHR 1.26, 95% CI 0.91–1.76, *P* = 0.17).

**Conclusions:**

Community-onset UTI due to EB demonstrating ESC-resistance is associated with a significantly increased hazard of recurrent UTI within 12 months compared to ESC-susceptible EB, even after adjusting for baseline factors that predispose patients to UTI recurrence. This association appears to be driven primarily by delayed or inappropriate treatment for the index ESC-R EB UTI.

## Background

Urinary tract infections (UTIs) are the most common bacterial infection among adults in the community [1], and there is significant bacterial resistance to first-line antibiotics used to treat UTIs in ambulatory settings [2]. In particular, there have been increasing reports of extended-spectrum cephalosporin-resistant (ESC-R) Enterobacteriaceae (EB) UTIs in the outpatient setting [3–7].

Recurrence is a frequent complication of UTI, with the risk for recurrence varying by population. In post-menopausal women, the risk for recurrence is as high as 80% in the following 12 months, with a mean of three recurrences during this period [8]. The risk is lower in men but remains notable with one study showing that the risk of recurrence within 12 months is 8% [9]. Known risk factors for recurrent UTI include female gender, sexual activity, diabetes mellitus, obesity, and anatomic abnormalities of the genitourinary tract [10].

Relatively little is known about the role that multidrug-resistant organisms (MDROs), and specifically ESC-resistance, play in recurrent UTIs. Prior studies have shown that patients with recurrent UTIs are more likely to ultimately develop an infection due to an MDRO, including ESC-R EB, likely related to repeated antibiotic exposures [5, 11]. However, it is unknown whether an infection due to an MDRO is in itself more likely to lead to recurrent UTI. To our knowledge, there are no prior studies that have evaluated whether an index UTI due to an ESC-R or extended-spectrum beta-lactamase (ESBL)-producing EB organism is associated with increased risk for UTI recurrence. Therefore, in this study, we sought to determine the association between community-onset ESC-R EB UTI and time to recurrent UTI.

## Methods

### Study design and setting

A retrospective cohort study was performed at two Emergency Departments (EDs) and a network of outpatient practices within the University of Pennsylvania Health System (UPHS), as follows: [1] the ED at the Hospital of the University of Pennsylvania (HUP), a 776-bed quaternary care medical center; [2] the ED at Penn Presbyterian Medical Center (PPMC), a 331-bed academic medical center, and [3] a network of 246 primary care physicians at over 50 community and hospital-based practices.

### Study population

The initial source population was composed of all patients presenting to an ED or outpatient practice who had a urine culture positive for EB between December 21, 2010 and April 22, 2013. Potentially eligible patients were identified through the HUP Clinical Microbiology Laboratory, which processes all cultures from HUP, PPMC, as well as > 90% of urine cultures from UPHS outpatient practices. A patient was designated as having a community-onset urine culture if it was obtained in the ED, outpatient practices, or within 72 h of hospital admission. Subsequently, patients were excluded if they were < 18 years-old, expired during the follow-up period, were a long-term care facility resident, or had a physician who failed to consent. The remaining subjects were eligible and were approached for consent. Those with an ESC-R EB organism on urine culture were approached first, and then a random selection of those with ESC-S EB organism on urine culture were approached in an equal number as those with ESC-R EB. After consenting, only patients with a true UTI were included as we sought to identify outcomes associated with ESC-R EB UTI rather than urinary colonization. A urine culture was considered indicative of an infection based on Centers for Disease Control and Prevention (CDC)/National Healthcare Safety Network (NHSN) criteria [12], which was determined via medical record review performed by an infectious diseases-trained physician (J.H.H.).

Exposed patients were defined as those with an EB UTI demonstrating resistance to an ESC (i.e., ceftriaxone or cefotaxime minimum inhibitory concentration [MIC] > 1 mg/L) according to Clinical and Laboratory Standards Institute (CLSI) criteria [13]. Unexposed patients were those who had a UTI with ESC-susceptible EB during the study period (i.e., ceftriaxone and cefotaxime MICs ≤1 mg/L). Unexposed patients were randomly selected from among all patients with ESC-susceptible EB UTIs using a computerized random number generator and were matched with exposed patients in a 1:1 ratio based on study year. This initial ESC-R or ESC-susceptible UTI (the exposure of interest) was designated as the “index UTI”. Each patient was included as a subject only once. The study was approved by the institutional review board of the University of Pennsylvania.

### Outcome

The primary outcome was time to first recurrent UTI. Modified CDC/NHSN criteria were used to confirm recurrent UTI [12]; our criteria relied on standard symptoms and signs of UTI along with pyuria on urinalysis but did not require a repeat urine culture, since urine cultures are infrequently sent in the outpatient setting. Further, urine cultures are more likely to be sent for patients with a history of ESC-R EB and would thus bias the analysis of recurrence. The outcome was determined via electronic medical record review by an infectious diseases-trained physician (J.A.A.). The electronic medical record system captures all outpatient, ED, and inpatient visits within the health system. The first clinical episode to meet the criteria for UTI was considered the first recurrent UTI following the index UTI. The outcome was assessed through 12 months following the index UTI.

### Data collection

Data on exposed and unexposed patients were abstracted from the UPHS electronic medical record system. Information was collected on demographics (e.g., age, gender, race), comorbidities (e.g., diabetes, malignancy, hemodialysis), urologic disorders (e.g., prior UTIs, urinary catheters), recent skilled nursing facility (SNF) or hospital stay, culture location (ED, inpatient, or outpatient practice), and all inpatient and outpatient antibiotic therapy that was prescribed in the 6 months preceding the UTI and in the 14 days following UTI diagnosis.

### Susceptibility testing of Enterobacteriaceae isolates

Susceptibility testing of EB isolates was performed by the HUP Clinical Microbiology Laboratory. All isolates identified from study subjects were tested as part of routine care for susceptibility to antibiotics using the semi-automated Vitek 2 identification and susceptibility system (bioMerieux, Inc., Durham, NC). Updated MIC breakpoints for ceftriaxone and cefotaxime were used without confirmatory ESBL testing according to CLSI guidelines [13].

### Statistical analysis

Exposed and unexposed patients were characterized by potential confounders, such as demographics, comorbidities, and prior antibiotic use. For these paired data, continuous variables were compared using the Wilcoxon signed rank test, and categorical variables were compared using the McNemar test. Bivariable Cox proportional hazard regression was used to examine the relationship between ESC-R EB UTI and time to recurrent UTI; the standard error was adjusted for clustering by matched pair. Patients were censored at the time of a recurrent UTI, death, or end of follow-up. Variables from bivariable analyses with *P* values < 0.20 or confounders of the primary association were considered for inclusion in the final multivariable model. Variables were added based on biologic plausibility. Variables were retained in the final model if they were confounders or if they had a *P* value of < 0.05 in the multivariable Cox model. A hazard ratio (HR) and 95% confidence interval (CI) were calculated to evaluate the strength of any association. All analyses were performed using STATA v.14.0 (StataCorp, College Station, Texas).

### Secondary analyses

In secondary exploratory analyses, we evaluated whether the association between ESC-R EB index UTI and time to recurrent UTI was impacted by prompt administration of antibiotics for the index UTI. Specifically, we evaluated the impact of inappropriate initial antibiotic therapy (IIAT). IIAT was binary and defined as failure to receive an antibiotic to which the organism was susceptible within 48 h of index urine culture collection.

## Results

### Study population

There were 2009 unique subjects who grew an Enterobacteriaceae species on a urine culture from an outpatient visit, ED visit, or within 72 h of hospital admission during the study period. After applying exclusion criteria, there were 887 subjects who were eligible. Of these 887 potential subjects, 574 (65%) consented to participate in the study. Of these, 151 had an ESC-R EB on urine culture that was consistent with true UTI (rather than colonization) and were thus the final “exposed” group. One hundred fifty-one patients with community-onset UTI due to an ESC-susceptible EB were then matched to the exposed patients based on study year and comprised the final “unexposed” group.

Within this cohort of 302 patients, the median age was 56 years (interquartile range [IQR], 37–68), and 62 (21%) were men. With the index UTI, 217 (72%) patients presented to an outpatient practice, while 85 (28%) patients presented to the ED. Baseline characteristics of the source cohort are shown in Table 1 stratified by exposure status.

**Table 1:** Baseline characteristics of the study cohort stratified by exposure status

Variable	ESC-S EB index UTI (unexposed)^a^	ESC-R EB index UTI (exposed)	*P* value
Demographics			
Age in years (median, IQR)	49 (27–64)	60 (46–70)	<0.01
Female gender	132 (87%)	108 (72%)	<0.01
Nonwhite race	81 (54%)	78 (52%)	0.73
Culture obtained in outpatient clinic	119 (79%)	89 (59%)	<0.01
Culture obtained in ED	29 (19%)	54 (36%)	<0.01
Culture obtained within 72 h of inpatient admission	3 (2%)	8 (5%)	0.23
Comorbidities/Exposures			
UTI in prior 6 months	57 (38%)	65 (43%)	0.37
Urinary catheter	9 (6%)	27 (18%)	<0.01
Surgery in prior 6 months	21 (14%)	36 (24%)	0.04
Prostate disease (if male)	6 (32%)	20 (47%)	>0.99
Liver disease^b^	1 (1%)	8 (5%)	0.04
Respiratory disease^c^	17 (11%)	29 (19%)	0.06
Diabetes mellitus	14 (9%)	31 (21%)	0.01
History of hemodialysis	1 (1%)	5 (3%)	0.22
Malignancy	11 (7%)	28 (19%)	<0.01
Prior renal transplantation	6 (4%)	13 (8%)	0.11
Rehabilitation or SNF stay in prior 6 months	3 (2%)	9 (6%)	0.11
Hospitalization in prior 6 months	35 (23%)	66 (44%)	<0.01
Antibiotic exposures^d^			
Any antibiotic	84 (56%)	94 (62%)	0.24
First-generation cephalosporin	8 (5%)	18 (12%)	0.04
Extended-spectrum cephalosporin	4 (3%)	19 (13%)	<0.01
Extended-spectrum penicillin	5 (3%)	12 (8%)	0.07
Amoxicillin/clavulanate or ampicillin/sulbactam	5 (3%)	11 (7%)	0.11
Piperacillin-tazobactam	0 (0%)	1 (1%)	>0.99
Fluoroquinolone	28 (19%)	41 (27%)	0.07
Aztreonam	0 (0%)	1 (1%)	>0.99
Carbapenem	1 (1%)	5 (3%)	0.02
Aminoglycoside	1 (1%)	3 (2%)	0.62
Nitrofurantoin	26 (17%)	28 (19%)	0.78
TMP-SMX	19 (13%)	36 (24%)	0.02
Fosfomycin	1 (1%)	2 (1%)	>0.99
Severity of index UTI			
Pyelonephritis at diagnosis	18 (12%)	44 (29%)	<0.01
BSI at diagnosis	3 (2%)	6 (4%)	0.51
Admitted to hospital	12 (8%)	30 (20%)	<0.01
Causative organism of index UTI			
*Escherichia coli*	116 (77%)	112 (74%)	0.59
*Klebsiella* species	18 (12%)	20 (13%)	0.72
*Enterobacter* species	12 (8%)	14 (9%)	0.69
*Citrobacter* species	3 (2%)	1 (1%)	0.63
*Proteus* species	2 (1%)	4 (3%)	0.69

^a^Data are presented as numbers (percentages) except where noted

^b^Hepatitis or cirrhosis

^c^COPD or chronic bronchitis

^d^Receipt in the 6 months prior to index EB UTI presentation

Abbreviations: *BSI* bloodstream infection, *CI* confidence interval, *ESC-R* extended-spectrum cephalosporin-resistant, *ESC-S* extended-spectrum cephalosporin-susceptible, *ED* Emergency Department, *IQR* interquartile range, *SNF* skilled nursing facility, *TMP-SMX* trimethoprim-sulfamethoxazole, *UTI* urinary tract infection

### Association of ESC-R EB UTI with time to recurrent UTI

Within the entire cohort, 163 (54%) patients developed a recurrent UTI within 12 months of the index UTI. The median time to recurrent UTI was 69 days (IQR 25–183). There was documented follow-up within the UPHS system for 286 (95%) of the patients, through to the time of recurrent UTI, death, or 12 months of follow-up.

Among the 163 recurrent UTIs, only 32 had an accompanying urine culture at the time of the recurrent UTI diagnosis. Among those 32 with urine cultures, 16 were in the ESC-R EB index UTI group, and 16 were in the ESC-S EB index UTI group. All of the recurrent UTIs with an accompanying urine culture, in both groups, were due to the same organism as was identified on the index urine culture. Eleven (34%) were due to an ESC-R EB organism, and 2 (6%) were due to a carbapenem-resistant EB organism; all of these multidrug-resistant EB UTI recurrences occurred in subjects who had had an ESC-R EB index UTI.

**Fig. 1:**
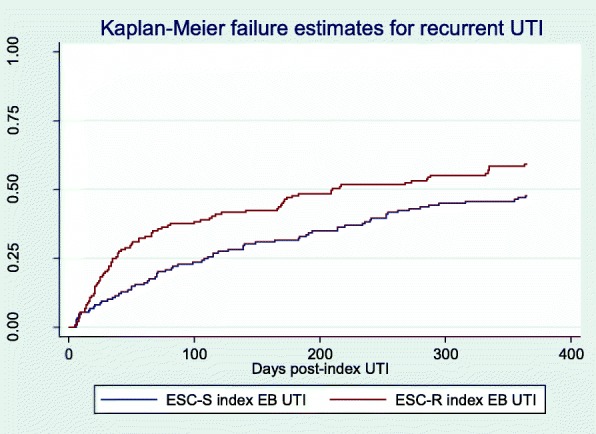
Time to recurrent UTI stratified by exposure. Legend: Abbreviations: EB, Enterobacteriaceae; ESC-S, extended-spectrum cephalosporin-susceptible; ESC-R, extended-spectrum cephalosporin-resistant; UTI, urinary tract infection

In the unadjusted analysis, we found that ESC-R EB UTI was associated with an increased hazard of recurrent UTI (hazard ratio [HR] 1.46, 95% confidence interval [CI] 1.08–1.98, *P* value = 0.02). The unadjusted Kaplan-Meier survival curve is shown in Fig. 1 (log rank test *P* value = 0.02). In the final multivariable model (Table 2), ESC-R EB UTI remained significantly associated with an increased hazard of recurrent UTI (adjusted HR [aHR] 1.39, 95% CI 1.01–1.91, *P* value = 0.04). Other factors that were associated with an increased hazard of recurrence included a history of UTI in the 6 months prior to the index UTI (aHR 1.59, 95% CI 1.17–2.15, *P* value < 0.01) and a urinary catheter at the time of the index UTI (aHR 1.59, 95% CI 1.06–2.38, *P* value 0.03). There was a decreased hazard of recurrent UTI associated with a SNF stay in the 6 months prior to the index UTI (aHR 0.38, 95% CI 0.19–0.77, *P* value = 0.01). Of note, presentation with pyelonephritis or bacteremia was not associated with a significantly increased hazard of recurrent UTI (pyelonephritis HR 0.96, 95% CI 0.65–1.43; bacteremia HR 1.09, 95% CI 0.45–2.68).

**Table 2:** Multivariable Cox proportional hazards regression model of recurrent UTI

Variable	aHR (95% CI)	*P* value
*ESC-R status*	*1.39 (1.01–1.91)*	*0.04*
Prior UTI^a^	1.59 (1.17–2.15)	<0.01
Urinary catheter^b^	1.59 (1.06–2.38)	0.03
Recent SNF stay^a^	0.38 (0.19–0.77)	0.01

^a^Within the 6 months prior to the index UTI

^b^Catheter in place at the time of the index UTI diagnosis

Abbreviations: *aHR* adjusted hazard ratio, *ESC-R* extended-spectrum cephalosporin resistant, *CI* confidence interval, *SNF* skilled nursing facility, *UTI* urinary tract infection

### Treatment received for the index UTI

The most commonly prescribed antibiotics for the treatment of the index UTI were fluoroquinolones (100, 33%), nitrofurantoin (94, 31%), and trimethoprim-sulfamethoxazole (73, 24%). First-, third-, and fourth-generation cephalosporins were administered less frequently (11, 6, 11% respectively). Of note, a similar proportion of the exposed and unexposed subjects received first- or third-generation cephalosporins: 17 (11%) subjects received first-generation cephalosporins in both groups; 9 (6.0%) of the exposed and 8 (5.3%) of the unexposed subjects received a third-generation cephalosporin. More subjects in the exposed group received a fourth-generation cephalosporin (cefepime) than in the unexposed group (24 [17.7%] exposed and 7 [5.1%] unexposed).

Within the entire cohort, 158 patients (53%) experienced IIAT. When IIAT was incorporated into the final multivariable Cox model for recurrent UTI (Table 3), we found that ESC-R status was no longer significantly associated with an increased hazard for recurrence (aHR 1.26, 95% CI 0.91–1.76, *P* value = 0.17). IIAT, however, was significantly associated with an increased hazard of recurrence (aHR 1.47, 95% CI 1.04–2.06, *P* value = 0.03).

**Table 3:** Multivariable Cox proportional hazards regression model of recurrent UTI including inappropriate initial antibiotic therapy

Variable	aHR (95% CI)	*P* value
*ESC-R status*	*1.26 (0.91–1.76)*	*0.17*
Prior UTI^a^	1.53 (1.12–2.11)	0.01
Urinary catheter^b^	1.51 (0.99–2.32)	0.06
Recent SNF stay^a^	0.36 (0.17–0.75)	0.01
Inappropriate initial antibiotic therapy	1.47 (1.04–2.06)	0.03

^a^Within the 6 months prior to the index UTI

^b^Catheter in place at the time of the index UTI diagnosis

Abbreviations: *aHR* adjusted hazard ratio, *CI* confidence interval, *ESC-R* extended-spectrum cephalosporin-resistance, *SNF* skilled nursing facility, *UTI* urinary tract infection

Due to the unexpected finding that prior SNF stay was associated with a decreased hazard of UTI recurrence, we also secondarily explored this relationship. We found that there were 12 patients in the cohort with a recent SNF stay, of which 11 were evaluated in the ED for their index UTI, 7 were admitted, and 7 received intravenous cefepime as the initial antibiotic. When treatment with cefepime was added to the multivariable model of UTI recurrence, prior SNF stay was no longer significantly associated with UTI recurrence (aHR for recent SNF stay 0.46, 95% CI 0.18–1.18, *P* value = 0.11).

## Discussion

In this study, we found that patients who presented with a community-onset UTI due to an ESC-R organism had a significantly increased hazard of recurrent UTI within 12 months compared to those with an ESC-susceptible EB UTI. We also found that a greater proportion of patients with an ESC-R EB UTI experienced a delay in appropriate antibiotic initiation and that this impacted the relationship between ESC-R EB status and recurrence. More specifically, after accounting for IIAT, we found that the association between ESC-R status and recurrence was no longer significant, suggesting that the increased risk for recurrence with an ESC-R EB UTI may be related to the timing and selection of the treatment regimen.

To our knowledge, there are no prior studies that have specifically evaluated whether community-onset UTI with an ESC-R or ESBL-producing EB is associated with an increased risk for recurrence. There are prior studies demonstrating that with recurrent UTIs, there is increased risk for ultimately developing an ESC-R EB on urine culture [5, 11]. Our study suggests that there may be a cyclic relationship, where the development of a UTI with an ESC-R EB organism increases the risk for further recurrence, related primarily to inappropriate treatment of such organisms and an associated lack of cure.

In addition to ESC-R status, we also found in our primary analysis that the hazard of recurrent UTI was significantly increased in the setting of prior UTIs (specifically a UTI diagnosed within 6 months of the index UTI). This finding is consistent with prior literature that has demonstrated that with each additional UTI, there is an incremental increase in the risk for another future UTI [8]. We also found that the presence of a urinary catheter was associated with an increased hazard of recurrence. This too is consistent with prior literature which has shown that urinary catheters are a significant risk factor for UTI as they create a portal of entry for bacteria into the genitourinary tract and may allow organisms to persist in the setting of biofilm production [10].

There are potential limitations of our study. Misclassification is a concern in retrospective studies. However, both the exposure and outcome were validated through medical record review by infectious diseases-trained physicians, rather than relying on diagnostic or billing codes. The assessment of the outcome was limited to review of the UPHS medical record. Patients who experienced clinical failure and sought care from outside providers would not have been captured. However, we found that 95% of the cohort had documented follow-up in our medical system. Further, the secondary analysis evaluating antibiotic use (IIAT) is potentially biased by confounding by indication. As a result, our analysis of IIAT was limited to secondary exploratory analyses for hypothesis-generation. Finally, because the present study was conducted in a single healthcare system, the results may not be generalizable to other dissimilar institutions.

## Conclusions

The results of our study demonstrate that community-onset ESC-R EB UTIs are associated with an increased hazard of recurrent UTI compared to ESC-susceptible EB UTIs, even after adjusting for other factors that predispose to recurrence, and that this association may be related to the antibiotic regimen received for the index UTI. Further studies are needed to determine interventions that may reduce the risk of recurrence, including different antibiotic regimens and durations.

## Abbreviations

(aHR): Adjusted HR
(CDC): Centers for Disease Control and Prevention
(CI): Confidence interval
(CLSI): Clinical and Laboratory Standards Institute
(EB): Enterobacteriaceae
(EDs): Emergency Departments
(ESBL): Extended-spectrum beta-lactamase
(ESC-R): Extended-spectrum cephalosporin resistance
(HR): Hazard ratio
(HUP): Hospital of the University of Pennsylvania
(IIAT): Inappropriate initial antibiotic therapy
(IQR): Interquartile range
(MDROs): Multidrug-resistant organisms
(MIC): Minimum inhibitory concentration
(NHSN): National Healthcare Safety Network
(PPMC): Penn Presbyterian Medical Center
(SNF): Skilled nursing facility
(UPHS): University of Pennsylvania Health System
(UTIs): Urinary tract infections
